# Supplementary material for: Elevated glycolysis imparts functional ability to CD8+ T cells in HIV infection
Source: Life Sci Alliance. 2021 Sep 21;4(11):e202101081. doi: 10.26508/lsa.202101081 (PMC8473722; doi:10.26508/lsa.202101081)
Supplement: Supplementary file 1 [file LSA-2021-01081_TableS1.docx]

**Supplemental Table 1: Seahorse data of ex vivo CD8 + T cells PBMCCD8+ T cells**

**Metabolic status of overnight rested CD8+ T cells**

|  | **HIV uninfected** | **Early HIV** | **Early HIV Tx** | **Chronic HIV** | **Chronic HIV Tx** | **Viral Controller** |
| --- | --- | --- | --- | --- | --- | --- |
| **Baseline OCAR (pmol/min)** | 17.8±1.0 | 20.0±2.8 | 12.2±1.7 * | 22.4±2.0 | 19.5±1.4 | 26.4±3.1 * |
| **ATP linked OCAR (pmol/min)** | 18.0±0.8 | 19.6±2.5 | 12.0±1.5 ** | 23.1±1.8 | 22.5±1.5 * | 28.8±2.8 ** |
| **Spare OCAR Capacity (pmol/min)** | 54.7±7.0 | 104.0±35.0 | 27.6±6.5 ** | 88.9±12.1 | 98.2±16.7 | 76.1±14.3 |
| **Baseline Glycolysis (mpH/min)** | 7.4±0.9 | 12.5±0.9 *** | 8.1±0.8 | 7.0±0.8 | 6.1±0.6 | 10.3±0.8 |
| **Maximum Glycolysis (mpH/min)** | 14.7±1.8 | 25.3±4.4 * | 10.4±1.5 | 22.8±3.2 | 15.8±1.4 | 20.5±2.1 |
| **Spare Glyolytic Capacity (mpH/min)** | 12.0±0.9 | 17.7±2.4* | 7.9±1.1 ** | 13.5±1.6 | 9.8±0.6 | 13.8±1.3 |

Reported as values ± standard error of mean; significance when compared to HIV uninfected

*=p<.05; **=p<.01; ***=p<.001; ****=P<.0001; na=not applicable

**Metabolic status of overnight stimulated CD8+ T cells**

|  | **HIV uninfected** | **Early HIV** | **Early HIV Tx** | **Chronic HIV** | **Chronic HIV Tx** | **Viral Controller** |
| --- | --- | --- | --- | --- | --- | --- |
| **Baseline OCAR (pmol/min)** | 29.2±1.3 ****/na | 27.0±3.8 | 22.0±1.9 **/* | 34.4±2.7 ***/ | 40.1±3.2 ****/** | 29.2±1.8 |
| **ATP linked OCAR (pmol/min)** | 29.5±1.5 ****/na | 25.4±3.6 | 21.9±1.8 **/* | 32.7±2.8**/ | 40.2±3.3 ****/** | 27.9±2.2 |
| **Spare OCAR Capacity (pmol/min)** | 84.6±9.1 */na | 86.7±17.5 | 61.2±8.8**/* | 92.2±14.4 | 103.8±12.8 | 63.6±6.7 |
| **Baseline Glycolysis (mpH/min)** | 18.6±1.0****/na | 25.9±3.8 ***/ | 15.6±1.2 ****/ | 20.2±1.9 ****/ | 20.1±1.4**** / | 20.0±1.4 ****/ |
| **Maximum Glycolysis (mpH/min)** | 38.2±2.0 ****/na | 50.0±8.2 | 25.2±2.7 ****/** | 40.6±4.7 ***/ | 42.5±3.7 ****/ | 37.9±4.2 **/ |
| **Spare Glyolytic Capacity (mpH/min)** | 31.4±2.2 ****/na | 41.5±6.6 */ | 20.4±2.1 ****/** | 31.6±3.2 ****/ | 31.1±2.4 ****/ | 32.9±3.4 ****/ |

Significance symbols reported as: compared to autologous rested cells/compared to pre-stimulated HIV uninfected cells
